# The Antidiabetic Effect of Low Doses of *Moringa oleifera* Lam. Seeds on Streptozotocin Induced Diabetes and Diabetic Nephropathy in Male Rats

**DOI:** 10.1155/2015/381040

**Published:** 2015-01-05

**Authors:** Abdulrahman L. Al-Malki, Haddad A. El Rabey

**Affiliations:** ^1^Biochemistry Department, Faculty of Science, King Abdulaziz University, Jeddah, Saudi Arabia; ^2^Genetic Engineering and Biotechnology Institute, Sadat City University, P.O. Box 79, Sadat City, Monufia, Egypt

## Abstract

The antidiabetic activity of two low doses of *Moringa* seed powder (50 and 100 mg/kg body weight, in the diet) on streptozotocin (STZ) induced diabetes male rats was investigated. Forty rats were divided into four groups. The diabetic positive control (STZ treated) group showed increased lipid peroxide, increased IL-6, and decreased antioxidant enzyme in the serum and kidney tissue homogenate compared with that of the negative control group. Immunoglobulins (IgA, IgG), fasting blood sugar, and glycosylated hemoglobin (HbA_1c_) were also increased as a result of diabetes in G2 rats. Moreover albumin was decreased, and liver enzymes and *α*-amylase were not affected. In addition, the renal functions and potassium and sodium levels in G2 were increased as a sign of diabetic nephropathy. Urine analysis showed also glucosuria and increased potassium, sodium, creatinine, uric acid, and albumin levels. Kidney and pancreas tissues showed also pathological alteration compared to the negative control group. Treating the diabetic rats with 50 or 100 mg *Moringa* seeds powder/kg body weight in G3 and G4, respectively, ameliorated the levels of all these parameters approaching the negative control values and restored the normal histology of both kidney and pancreas compared with that of the diabetic positive control group.

## 1. Introduction

Diabetes mellitus (DM) is a metabolic disorder that threatens the life of World populations leading to hyperglycemia which is the major cause of diabetic complications, such as retinopathy, nephropathy, and neuropathy [1, 2]. Diabetic nephropathy (DN) is structural abnormalities revealing hypertrophy of both glomerular and tubular elements; increase in the thickness of glomerular basement membranes, progressive accumulation of extracellular matrix components, early increase in the glomerular filtration rate with intraglomerular hypertension, subsequent proteinuria, systemic hypertension, and eventual loss of renal function are also signs of diabetic nephropathy [3].

*Moringa oleifera* Lamarck (*Moringa*) is the cultivated species of the genus* Moringa* of the family Moringaceae. Several health benefits were reported as a result of supplementation with* Moringa* leaves or seeds or their extract [4–6].* M. oleifera* is described as the miracle tree, tree of life, and God's Gift to man [7].

*Moringa* root wood reduced the elevated urinary oxalate and lowered the deposition of stone forming constituents in the kidneys of calculogenic rats as a result of ethylene glycol treatment [8].* Moringa* improved nutrition, boosted food security, fostered rural development support sustainable land care, and foraged for livestock [9].* Moringa* ameliorated liver fibrosis in rats and reduces liver damage and symptoms of liver fibrosis, decreased the CCl_4_-induced elevation of serum aminotransferase activities and globulin level, and reduced the elevated hepatic hydroxyproline content and myeloperoxidase activity [5].

The antioxidant and antidiabetic activity of aqueous extract of* Moringa* leaves indicated potential benefits as a potent antidiabetic in streptozotocin induced diabetic albino rats [6].* Moringa* crude extract was also a good scavenger for nitric oxide radicals and has a potential source of natural antioxidant [10].* Moringa* has also nutraceutical uses and is used in treatment of hypercholesterolemia and hyperglycemia, and also, as a nutritional supplementation, it can be prescribed as food appendage for coronary artery disease patients along with their regular medicines [11].* Moringa* also increased wound healing of normal and dexamethasone suppressed wound in rats [12].

In spite of the medical benefits of* Moringa*, the high concentrations had toxologic effects in mice and rats [10, 13].

This study aimed at testing the ameliorative effect of two low doses of* Moringa* seeds powder (50 and 100 mg/kg body weight) on type I diabetes and treating diabetic nephropathy of streptozotocin induced diabetic male rats.

## 2. Materials and Methods

### 2.1. *Animals* and Diet

Forty adult male Albino rats weighing 180 to 200 g were used in this study. The animals were kept for two weeks as an acclimatization period prior to the start of the experiment. They were housed 5/cage and received normal basal diet and tap water* ad libitum* in a constant environment (room temperature 28 ± 2°C, room humidity 60 ± 5%) with a 12 h light and 12 h dark cycle.

The conventional animal basal diet was obtained from a grain mill in Jeddah. Each 100 gm consists of the following: 12% protein (17.14 g 70% casein), 4 g corn oil (4% fat), 0.3 g methionine (0.3%), 0.2 g choline chloride (00.2%), 4 g minerals (4% minerals), 1 g vitamin mixture (1% vitamin), 4 g cellulose (4% fiber), and 69.36 g corn starch (69.36%). The basal diet was stored in a dry place out of direct sunlight.

### 2.2. Experiment Design

All animal experiments were carried out under protocols approved by the Institutional Animal House of the University of King Abdulaziz, Jeddah, Saudi Arabia. The animals were divided into 4 groups each consisting of 10 rats. The first group (G1) received a single tail vein injection of 0.1 mol/L citrate buffer only. The other 30 rats were intravenously injected with freshly prepared streptozotocin (60 mg/kg body weight) in a 0.1 mol/L citrate buffer (pH 4.5), after fasting for 12 h [14]. After five days of injection, rats with blood glucose higher than 200 mg/dL were considered as being diabetic in the fasting state. Rats with blood glucose lower than 200 mg/dL were excluded from the study. The study was started one week after STZ injection. The 30 diabetic rats were randomly divided into 3 groups: the second group (G2) received only STZ and was fed normal basal diet. The third group was treated with low dose of* Moringa* seed powder (50 mg/kg b.w.) in the diet. The fourth group (G4) was treated with 100 mg/kg b.w.* Moringa* seeds powder in the diet. Treatment was continued for 4 weeks.

At the end of the experiment, animals were sacrificed using ether anaesthesia. Kidneys and pancreas were dissected and rinsed in saline buffer (0.9% NaCl).

### 2.3. Kidney Homogenate Preparation

All steps were achieved at 4°C. Kidney tissue was cut into small pieces and washed with phosphate-buffered saline and then grinded in a homogenization buffer consisting of 0.05 M Tris-HCl pH 7.9, 25% glycerol, 0.1 mM EDTA, and 0.32 M (NH4)_2_SO_4_ and containing a protease inhibitor tablet (Roche, Germany). The lysates were homogenized on ice using a Polytron homogenizer. The solution was sonicated in an ice bath to prevent overheating for 15 seconds followed by 5-minute centrifugation at 12000 rpm and 4°C. The supernatant was aliquoted and stored at −80°C. The homogenate was used for determination of the activity of antioxidant enzymes, level of lipid peroxidation, and level of IL-6. The other kidney from each group was used for histopathological examination.

### 2.4. Blood Sampling and Biochemical Analysis

Blood samples of rats were centrifuged at 2,000 g for 10 minutes at 4°C and aliquoted for the respective analytical determinations.

### 2.5. Determination of Lipid Peroxide and Antioxidant Enzymes Activity

Lipid peroxide was estimated by measuring malondialdehyde (MDA) and the activity of catalase, reduced glutathione (GSH), and superoxide dismutase (SOD) in the serum and in the kidney tissue homogenate using the specified kits from Biodiagnostic Chemical Company (Egypt) according to the instructions of the supplier.

### 2.6. Immunoglobulins (IgG, IgA)

The IgG immunoglobulins were estimated using Rat IgG kit, Immunoperoxidase Assay for Determination of IgG in Rat Samples from Genway Biotech (USA) according to the instruction of the supplier.

The IgA immunoglobulins were estimated using Rat IgA kit, Immunoperoxidase Assay for Determination of IgA in Rat Samples from Genway Biotech (USA) according to the instructions of the supplier.

### 2.7. Determination of IL-6

IL-6 concentration in the serum and in the kidney homogenate was determined using Rat IL-6 Immunoassay kit from R&D Systems Inc. (USA) according to the instructions of the supplier.

### 2.8. Glycosylated Hemoglobin

Glycosylated hemoglobin (HbA_1c_) was estimated using Glycohemoglobin Reagent Set from Pointe Scientific Inc. (USA) according to the instructions of the suppliers.

### 2.9. Liver Enzymes

Serum alanine aminotransferase (ALT) activity was estimated using the modified kinetic method of Srivastava et al. [15] using a kit supplied by Human, Germany, according to the instructions of the supplier. Serum aspartate aminotransferase (AST) activity was estimated using the modified kinetic method of Schumann and Klauke [16] using a kit supplied by Human, Germany, according to the instructions of the supplier. Serum alkaline phosphatase (ALP) activity was estimated using the modified kinetic method of Tietz and Shuey [17] using a kit supplied by Human, Germany, according to the instructions of the supplier.

### 2.10. Serum *α*-Amylase Determination

Serum *α*-amylase activity was estimated using a kit from Human (Germany) according to the instructions of the supplier.

### 2.11. Fasting Blood Sugar Determination

Fasting blood sugar was estimated using glucose kit from Human (Germany) according to the instructions of the supplier.

### 2.12. Serum Albumin Determination

Serum albumin concentration was estimated using Albumin Assay Kit from Sigma-Aldrich (USA) according to the instructions of the supplier.

### 2.13. Renal Functions

Serum uric acid, serum creatinine, and serum urea nitrogen were estimated using Human Kits (Germany) according to the instructions of the supplier.

### 2.14. Serum Electrolytes

Sodium and potassium ions were estimated in serum using the specified kit from Human (Germany) according to the instructions of the supplier.

### 2.15. Analysis of Urine Parameters

Urine samples were collected in individual metabolic cages for 24 h, the day before the end of treatment. Sugar, albumin, creatinine, urea, potassium, and sodium levels were estimated using the specified kits according to the instructions of the supplier.

### 2.16. Physiological Parameters

The following biological parameters were estimated.

- Food intake and water consumption were calculated every week.
- Total body weight: rats were weighed every week.
- Food intake (FI) body weight gain (BWG) and food efficiency ratio (FER) were calculated.
- Organ weight and relative organ weight: heart, liver, right kidney, left kidney, left testis, and right testis were weighed after dissection and the relative organ weight was calculated by dividing the organ weight on the total body weight of each rat and then multiplied by 100.

### 2.17. Histopathological Examination

Renal and pancreatic tissues were collected after animal sacrifice, fixed in 10% formalin, processed routinely, and embedded in paraffin. 5 *μ*m thick sections were prepared and stained with hematoxylin and eosin (H&E) dye for microscopic investigation [18]. The stained sections were examined and photographed under a light microscope.

### 2.18. Statistical Analysis

Values were analyzed using SPSS program to calculate the *t*-test and the mean ± SD (standard error) and then analyzed using one way analysis of variance (ANOVA) using Duncan's Multiple Range Test [19] to test the significance at *P* < 0.05 and to identify the best treatment group.

## 3. Results

### 3.1. Lipid Peroxide

Table 1 shows the effect of treating diabetic rats with* Moringa* for 4 weeks on lipid peroxidation in the serum and kidney tissue homogenate. The mean value of lipid peroxide in the diabetic positive control (STZ treated) group was high, that is, significantly increased compared with that of the negative control group in both plasma and kidney tissue homogenate. Treating these diabetic rats with 50 or 100 mg* Moringa* seeds powder/kg body weight significantly reduced the lipid peroxide compared with the positive control group. Treating diabetic rats with lower dose of* Moringa* seeds powder in G3 (50 mg/kg body weight) decreased lipid peroxide in the serum more than the 100 mg* Moringa* seeds powder treated group in G4. In contrast, the MDA in kidney tissue homogenate in G4 was decreased compared to that in the G3.

### 3.2. Antioxidants Enzymes

The result of treating diabetic rats with* Moringa* for 4 weeks on antioxidant enzymes in the serum and kidney tissue homogenate is given in Table 1. The mean values of catalase, superoxide dismutase, and glutathione reduced in the positive control group were high, that is, significantly (at *P* < 0.001) decreased compared with that of the negative control group in both plasma and kidney tissue homogenate. Treating diabetic rats with 50 or 100 mg* Moringa* seeds powder/kg body weight significantly (at *P* < 0.001) increased all antioxidant enzymes in the serum and kidney tissue homogenate. In G4, the mean value of the three antioxidant enzymes was higher than that of the G3.

### 3.3. Immunoglobulins

The mean values of IgG and IgA immunoglobulins were significantly (at *P* < 0.001) increased in the diabetic rats (G2) compared with that of the negative control group. Treating diabetic rats with* Moringa* for 4 weeks significantly (at *P* < 0.001) decreased IgG and IgA in the serum as shown in Table 2. The higher dose in G4 was more effective than that of the G3.

### 3.4. Interleukin-6 (IL-6)

Table 2 shows also the effect of treating diabetic rats with* Moringa* for 4 weeks on interleukin-6 (IL-6) in the serum and kidney tissue homogenate. The mean value of IL-6 in the positive control group was high, that is, significantly (at *P* < 0.001) increased compared with that of the negative control group in both plasma and kidney tissue homogenate. Treating these diabetic rats with 50 or 100 mg* Moringa* seeds powder/kg body weight in G3 and G4, respectively, significantly (at *P* < 0.001) decreased IL-6 values compared with the positive control group. Treating diabetic rats with the higher dose of* Moringa* seeds powder in G4 was more effective than that of G3.

### 3.5. Glycosylated Hemoglobin

To test the degree of success in controlling blood sugar with* Moringa*, the hemoglobin A_1c_ test was achieved. The percentage of HbA_1c_ was significantly (at *P* < 0.001) increased in the diabetic rats of the positive control group compared with that of the negative control group (Table 2). When the diabetic rats were treated with* Moringa*, the hemoglobin A_1c_ was significantly (at *P* < 0.001) decreased compared with the positive control in G2. Treating rats with 100 mg/kg body weight* Moringa* seed powder dose in G4 was more efficient than treating with the lower dose in G3.

### 3.6. Liver Enzymes

Table 3 shows the effect of treating diabetic rats with* Moringa* for four weeks on liver enzymes. The mean values of ALT, AST, and ALP were not significantly affected by diabetes. Treating diabetic rats in G3 and G4 with the two doses of* Moringa* under study did not affect the studied liver enzymes.

### 3.7. *α*-Amylase

Table 3 shows also that the mean value of *α*-amylase was not significantly increased due to STZ induced diabetes in the positive control group (G2). Treating these rats with different doses of* Moringa* in G3 and G4 nonsignificantly reduced the *α*-amylase.

### 3.8. Fasting Blood Sugar (FBS)

Table 3 shows also that the mean value of serum fasting blood sugar (FBS) was high, that is, significantly (at *P* < 0.001) increased in the STZ diabetic rats in the positive control group (G2). However, treating these rats with different doses of* Moringa* seeds powder for 4 weeks significantly (at *P* < 0.001) decreased the fasting blood sugar in the serum of G3 and G4 groups, although being higher than the negative control values.

### 3.9. Kidney Functions

The mean value of blood urea nitrogen (BUN), uric acid, and creatinine in the serum of G2 was significantly (at *P* < 0.001) increased compared with that of the negative control group (G1) as shown in Table 4. Treating these diabetic rats with 50 or 100 mg* Moringa* seeds powder/kg body weight in G3 and G4, respectively, significantly (at *P* < 0.001) increased and ameliorated all kidney functions parameters under study compared with that of the positive control group. The higher dose of* Moringa* seeds powder in G4 was more effective than that of G3.

### 3.10. Serum Electrolytes

Table 4 shows also that the mean value of sodium and potassium in the serum of G2 was significantly (at *P* < 0.001) increased compared with that of the negative control group (G1). Treating these diabetic rats with 50 or 100 mg* Moringa* seeds powder/kg body weight in G3 and G4, respectively, significantly (at *P* < 0.001) ameliorated both sodium and potassium levels compared with those of the positive control group. The lower dose of* Moringa* seeds powder in G3 was more effective than that of the higher dose in G4.

### 3.11. Urine Analysis

Table 5 shows that all mean values of glucose, urea nitrogen, creatinine, albumin, sodium, and potassium in urine of G2 were significantly (at *P* < 0.001) increased compared with that of the negative control group (G1). Treating these diabetic rats with 50 or 100 mg* Moringa* seeds powder/kg body weight in G3 and G4, respectively, significantly (at *P* < 0.001) ameliorated levels of all these parameters compared with that of the positive control group. Except urea nitrogen which was highly ameliorated with the lower dose of* Moringa* seeds powder in G3 than that of the higher dose in G4, the other parameters were more ameliorated by treating with the higher dose in G4 than that of G3.

### 3.12. Total Body Weight

Table 6 shows that there is no significant difference between the initial body weights, whereas the mean values of body weight in G2 after the first week, the second week, the third week, and the fourth week were significantly (at *P* < 0.001) decreased compared with that of the negative control group (G1). Treating these diabetic rats with 50 or 100 mg* Moringa* seeds powder/kg body weight in G3 and G4, respectively, significantly (at *P* < 0.001) increased the body weight approaching that of the negative control group.

### 3.13. Water Consumption

Table 7 shows that the mean value of water consumption was high, that is, significantly (at *P* < 0.001) increased in the first, second, third, and fourth weeks as a result of STZ induced diabetes in G2 compared with that of the negative control group (G1). Treating diabetic rats with 50 or 100 mg* Moringa* seeds powder/kg body weight in G3 and G4, respectively, significantly (at *P* < 0.001) decreased water consumption in all weeks approaching the normal consumption of G1.

### 3.14. Physiological Parameters

Table 8 shows that the mean value of food intake (IF), body weight gain (BWG), body weight gain percentage (BWG%), food efficiency ratio (FER), and food efficiency ratio percentage (FER%) was significantly (at *P* < 0.001) increased, a result of STZ induced diabetes in G2 compared with that of the negative control group (G1). Treating these diabetic rats with 50 or 100 mg* Moringa* seeds powder/kg body weight in G3 and G4, respectively, significantly (at *P* < 0.001) decreased the mean values of these parameters approaching the normal values of G1.

### 3.15. Organs Weight

Table 9 shows that the mean value of heart, liver, right kidney, left kidney, right testis, and left testis weight was nonsignificantly increased or decreased as a result of STZ induced diabetes in G2 compared with that of the negative control group (G1). Treating these diabetic rats with 50 or 100 mg* Moringa* seeds powder/kg body weight in G3 and G4, respectively, also nonsignificantly ameliorated the weight of these organs approaching the normal weights of G1.

### 3.16. Relative Organs Weight

Table 9 shows also that the mean value of the relative heart, liver, right kidney, left kidney, right testis, and left testis weight was high, that is, significantly (at *P* < 0.001) increased as a result of STZ induced diabetes in G2 compared with that of the negative control group (G1). Treating these diabetic rats with 50 or 100 mg* Moringa* seeds powder/kg body weight in G3 and G4, respectively, significantly (at *P* < 0.05 or *P* < 0.01 or *P* < 0.001) decreased the relative weight of these organs approaching the normal relative weight of G1.

### 3.17. Pathology of Kidney

**Figure 1:**
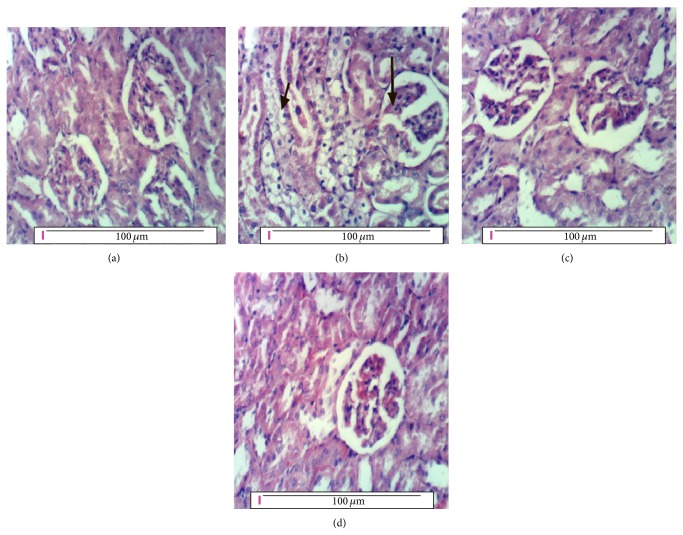
(a) Kidney of rat from the negative control group showing normal histological structure of renal parenchyma and glomeruli, (b) kidney of rat from the positive control group showing thickening of glomerular basement membrane and vacuolation of endothelial lining glomerular tuft (long arrow) and of epithelial lining renal tubules (short arrow), (c) kidney of diabetic rat treated with 50 mg/kg b.w.* Moringa* (G3) showing no histopathological changes, and (d) kidney of diabetic rat treated with 100 mg/kg b.w.* Moringa* (G4) showing no histopathological changes (H&E ×400).

Kidney of the negative control rats shows normal renal histological structure of renal parenchyma and glomeruli as shown in Figure 1(a).  Figure 1(b) shows kidney of rat from the positive control group with thickened glomerular basement membrane, vacuolated endothelial lining glomerular tuft, and vacuolated epithelial lining renal tubules. Treating diabetic rats with 50 mg/kg b.w. of* Moringa* seeds powder in G3 nearly restored the renal tissues to their normal histology with no histopathological changes as shown in Figure 1(c). In Figure 1(d), kidney tissue of diabetic rats in G4 treated with a higher dose of* Moringa* (100 mg/kg b.w.) restored the kidneys of diabetic rats to their normal case with no histopathological changes.

### 3.18. Pathology of Pancreas

**Figure 2:**
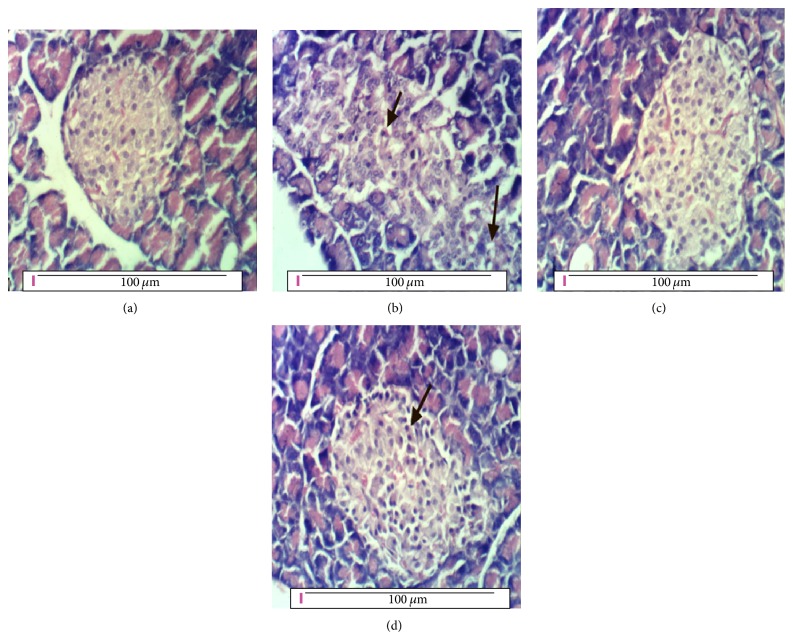
(a) Pancreas of rat from the negative control group showing no histopathological changes, (b) pancreas of rat from the diabetic positive control (G2) group showing necrosis and vacuolations of pancreatic acini and Langerhans islets cells (arrow), (c) pancreas of diabetic rat treated with 50 mg/kg b.w.* Moringa* (G3) showing no histopathological changes, and (d) pancreas of diabetic rat treated with 100 mg/kg b.w.* Moringa* (G4) showing no histopathological changes (H&E ×400).

Pancreas of the negative control rats shows the normal lobular histological structure of pancreatic acini and Langerhans islets cells as shown in Figure 2(a). Langerhans islets are interspersed among the pancreatic acini, as compact spherical masses with intact interlobular connective tissue and interlobular ducts.  Figure 2(b) shows pancreatic tissues of rat from the diabetic positive control group with necrosis and vacuolations of pancreatic acini and Langerhans islets cells. Treating diabetic rats with 50 mg/kg b.w of* Moringa* seeds powder in G3 nearly restored the pancreatic tissues to their normal histology with no histopathological changes as shown in Figure 2(c). In Figure 2(d), pancreatic tissue of diabetic rats in G4 treated with a higher dose of* Moringa* (100 mg/kg b.w.) restored its normal structure and histology with no pathological changes.

## 4. Discussion

Diabetes mellitus is metabolic disorders leading to hyperglycemia which later develops to micro- and macrovascular complications and becomes a major cause of death. The mechanism of STZ as diabetogenic agents is mediated by reactive oxygen species, since enhanced ATP dephosphorylation after streptozotocin treatment supplies a substrate for xanthine oxidase resulting in the formation of superoxide radicals, hydrogen peroxide, and hydroxyl radicals [20].

In the current study, induction of diabetes using streptozotocin in rats of the positive control group caused severe health problems illustrated in the increase in serum glucose and increase in glycosylated hemoglobin which is consistent with previous findings [20, 21]. STZ diabetes in G2 rats showed also an increase in lipid peroxidation and IL-6 and decreased catalase, SOD, and GSH activity in the serum and the kidney tissue homogenate compared with that of the negative control group. This result is consistent with previous investigations [22–24]. The concurrent treatment with* Moringa* ameliorated these parameters and nearly restored them to their normal levels. This curative effect is due to the active constituents present in* Moringa* seeds [22, 23]. The antioxidant activity of* Moringa* seed powder is due to its content of phenolics and flavonoids that have scavenging effect on the free radicals [22, 23]. Furthermore, Ghiridhari et al. [23] reported that medication with* M. oleifera* gives diabetic patients better glucose tolerance by increasing treatment time.* M. oleifera* contains three classes of phytochemicals, that is, glucosinolates such as glucomoringin, flavonoids such as quercetin and kaempferol, and phenolic acids such as chlorogenic acid; all of these classes have medicinal benefits [6, 7]. These three phytochemicals of* Moringa* possess antioxidant, hypoglycemic, hypotensive, antidyslipidemic, anticancer, and anti-inflammatory properties [25–27].

Interleukin-6 is a cytokine involved not only in inflammation and infection responses but also in the regulation of metabolic, regenerative, and neural processes [28]. However, the increase of IL-6 in G2 as a result of STZ induced diabetes and its decrease with the concurrent treatment with* Moringa* suggests that* Moringa* has antidiabetic activity. In addition, IL-6 has in addition to its immunoregulatory actions been proposed to affect glucose homeostasis and metabolism directly and indirectly by action on skeletal muscle cells, adipocytes, hepatocytes, pancreatic *β*-cells, and neuroendocrine cells [29].

The increase of immunoglobulins (IgA, IgG) and the increase of glycosylated hemoglobin (HbA_1c_) as a result of diabetes are consistent with the increase in IL-6 and other findings revealed positive correlation between these parameters [21, 30].* Moringa* treatment ameliorated these parameters and nearly restored them to the normal levels. This result is consistent with the antidiabetic activity of* Moringa* [22, 31]. On the other hand, liver enzymes and *α*-amylase were not affected as a result of diabetes in G2 or treating with* Moringa*.

The renal function analytes and potassium and sodium levels were increased as a result of diabetic nephropathy which is considered a major complication of diabetes [27, 32]. This result is supported by earlier studies [3]. Urine analysis of the STZ induced diabetes rats showed also glucosuria and increased potassium, sodium, creatinine, uric acid, and albumin levels in the positive control group as a result of diabetic nephropathy [6, 32], which is a major complication of diabetes [6, 27, 32]. Diabetic nephropathy is a clinical syndrome characterized by persistent albuminuria, progressive decline in the glomerular filtration rate (GFR), and elevated arterial blood pressure [3].

The renal tissues of the positive control diabetic rats showed also the symptoms of diabetic nephropathy (DN) illustrated in the hypertrophy of both glomerular and tubular elements and increase in the thickness of glomerular basement membranes [3]. Similarly, the pancreatic tissues of the diabetic rats in G2 showed necrosis and vacuolations of pancreatic acini and Langerhans islets cells as a result of STZ injection [6]. These histopathological alterations in the positive control group rats together with the other tests achieved to diagnose diabetes and afterwards diabetic nephropathy emphasized that diabetic nephropathy was grown as one of the diabetic complications.

Treating the diabetic rats with 50 or 100 mg* Moringa* seeds powder/kg body weight in G3 and G4, respectively, ameliorated the levels of all these parameters approaching the negative control values and restored the normal histology of both kidney and pancreas compared with that of the diabetic positive control group in G2. This result agrees with the findings of Ndong et al. [33] and Parikh et al. [34].

The antidiabetic activity of the higher dose of* Moringa* seeds powder (100 mg/kg b.w.) was more efficient than that of the lower dose (50 mg/kg b.w.). This result is consistent with that of Kumbhare et al. [10] who noticed that the radical scavenging effect was found to be increased with increasing concentrations.

Treating diabetic rats with 50 mg/kg b.w. of* Moringa* seeds powder in G3 and 100 mg/kg b.w. in G4 restored the normal renal function and histology of kidney and pancreas with no pathological changes. This result is consistent with other findings using* Moringa* aqueous extract [6].* Moringa* succeeded in controlling diabetic nephropathy such as other plants, for example, ginger [32], and using different materials such as ferulsinaic acid [27].

In conclusion, treated STZ induced diabetes male rats with the low doses of* Moringa* revealed a safe and an excellent antidiabetic activity due to its content of antioxidant compounds such as glucomoringin, phenols, and flavonoids and almost restored the diabetic rats to the normal healthy state. In addition, lower doses of* Moringa* under study may have greater medical benefits when used as food supplement for diabetic people's diet.

## Figures and Tables

**Table 1:** Effect of treating diabetic rats with *Moringa* seeds powder for 4 weeks on lipid peroxide and antioxidants enzymes in serum and kidney tissue homogenate.

Parameters	Statistics	G1 −ve control	G2 +ve control	G3 50 mg *Moringa *	G4 100 mg *Moringa *
Serum MDA nmol/mL	Mean ± SE LSD 0.05 = 0.180	2.19 ± 0.05^a^	6.14 ± 0.08^d^	3.50 ± 0.04^c^	4.36 ± 0.05^b^
	*t*-test		−32.16^***^	25.24^***^	24.18^***^

Tissue MDA nmol/g kidney tissue	Mean ± SE LSD 0.05 = 0.450	3.375 ± 0.11^a^	11.18 ± 0.26^d^	7.60 ± 0.02^b^	6.18 ± 0.05^c^
	*t*-test		−27.39^***^	12.74^***^	19.37^***^

Serum catalase U/L	Mean ± SE LSD 0.05 = 0.119	2.66 ± 0.05^a^	0.24 ± 0.01^c^	1.40 ± 0.03^d^	1.82 ± 0.02^b^
	*t*-test		44.18^***^	−30.49^***^	−54.30^***^

Tissue catalase U/g kidney tissue	Mean ± SE LSD 0.05 = 0.178	4.13 ± 0.11^a^	0.44 ± 0.00^c^	1.89 ± 0.02^d^	2.72 ± 0.02^b^
	*t*-test		32.29^***^	62.28^***^	−88.30^***^

Serum SOD U/mL	Mean ± SE LSD 0.05 = 14.698	664.20 ± 4.06^a^	352.00 ± 5.15^c^	446.55 ± 5.20^d^	577.15 ± 4.76^b^
	*t*-test		44.87^***^	−11.29^***^	−26.52^***^

Tissue SOD U/g kidney tissue	Mean ± SE LSD 0.05 = 10.575	1124.7 ± 4.79^a^	421.63 ± 1.95^d^	624.30 ± 2.34^c^	819.33 ± 3.86^b^
	*t*-test		109.45^***^	−69.70^***^	−93.80^***^

GSH U/mL	Mean ± SE LSD 0.05 = 1.369	26.11 ± 0.63^a^	9.80 ± 0.30^c^	15.50 ± 0.24^d^	18.88 ± 0.45^b^
	*t*-test		18.24^***^	−15.18^***^	−14.99^***^

GSH U/g kidney tissue	Mean ± SE LSD 0.05 = 3.215	64.30 ± 1.24^a^	27.50 ± 0.77^d^	36.63 ± 0.88^c^	50.01 ± 1.45^b^
	*t*-test		32.72^***^	−10.68^***^	−12.02^***^

Data are represented as mean ± SE. *t*-test values; ^***^ *P* < 0.001. ANOVA analysis: within each row, means with different superscript (a, b, c, or d) are significantly different at *P* < 0.05, whereas means superscripts with the same letters mean that there is no significant difference at *P* < 0.05. LSD: least significant difference.

**Table 2:** Effect of treating diabetic rats with *Moringa* seeds powder for 4 weeks on immunoglobulins, IL-6, and HbA_1c_.

Parameters mg/dL	Treatments statistics	G1 −ve control	G2 +ve control	G3 50 mg *Moringa *	G4 100 mg *Moringa *
IgG mg/dL	Mean ± SE LSD 0.05 = 8.048	534.00 ± 2.12^a^	753.00 ± 3.24^d^	636.17 ± 3.45^b^	579.83 ± 2.16^c^
	*t*-test		−52.40^***^	23.38^***^	36.29^***^

IgA mg/dL	Mean ± SE LSD 0.05 = 10.305	102.83 ± 3.47^a^	358.17 ± 2.57^d^	231.33 ± 3.14^b^	175.17 ± 4.26^c^
	*t*-test		−47.59^***^	29.23^***^	35.93^***^

Serum IL-6 pg/mL	Mean ± SE LSD 0.05 = 1.989	3.400 ± 0.44^a^	21.58 ± 1.07^d^	13.91 ± 0.60^b^	8.35 ± 0.29^c^
	*t*-test		−16.32^***^	5.46^***^	14.14^***^

Tissue IL-6 pg/g kidney tissue	Mean ± SE LSD 0.05 = 4.074	57.30 ± 2.04^a^	88.70 ± 1.67^d^	78.08 ± 0.46^b^	69.95 ± 0.37^c^
	*t*-test		−11.47^***^	6.05^***^	10.08^***^

HbA_1c_ DCCT%	Mean ± SE LSD 0.05 = 0.280	5.01 ± 0.07^a^	7.81 ± 0.13^d^	6.81 ± 0.07^b^	6.15 ± 0.04^c^
	*t*-test		−15.33^***^	5.42^***^	12.74^***^

Data are represented as mean ± SE. *t*-test values; ^***^ *P* < 0.001. ANOVA analysis: within each row, means with different superscript (a, b, c, or d) are significantly different at *P* < 0.05, whereas means superscripts with the same letters mean that there is no significant difference at *P* < 0.05. LSD: least significant difference.

**Table 3:** Effect of treating diabetic rats with *Moringa* seeds powder for 4 weeks on liver enzymes (ALT, AST, and ALP), *α*-amylase, fasting blood sugar, albumin, renal functions, and serum electrolytes.

Parameters	Statistics	G1 −ve control	G2 +ve control	G3 50 mg *Moringa *	G4 100 mg *Moringa *
ALT U/L	Mean ± SE LSD 0.05 = 4.199	26.33 ± 1.45^a^	27.50 ± 1.80^a^	25.83 ± 1.70^a^	26.66 ± 1.62^a^
	*t*-test		−0.44^NS^	1.74^NS^	0.46^NS^

AST U/L	Mean ± SE LSD 0.05 = 9.452	27.00 ± 1.15^a^	25.83 ± 1.301^a^	25.66 ± 1.05^a^	26.50 ± 0.95^a^
	*t*-test		0.97^NS^	0.09^NS^	−0.37^NS^

ALP U/L	Mean ± SE LSD 0.05 = 29.741	114.17 ± 5.35^a^	109.50 ± 4.21^a^	111.83 ± 5.14^a^	92.00 ± 16.57^a^
	*t*-test		0.58^NS^	−0.40^NS^	0.91^NS^

*α*-Amylase U/L	Mean ± SE LSD 0.05 = 12.986	47.16 ± 4.52^a^	50.00 ± 3.58^a^	48.00 ± 5.26^a^	47.66 ± 3.77^a^
	*t*-test		−0.62^NS^	0.35^NS^	0.33^NS^

Fasting blood sugar (FBS) mg/dL	Mean ± SE LSD 0.05 = 7.375	87.66 ± 2.76^a^	266.50 ± 2.17^d^	174.17 ± 1.86^b^	148.83 ± 2.44^c^
	*t*-test		−39.06^***^	45.53^***^	29.01^***^

Albumin g/dL	Mean ± SE LSD 0.05 = 0.311	3.950 ± 0.13^a^	1.400 ± 0.09^d^	2.133 ± 0.06^c^	2.76 ± 0.07^b^
	*t*-test		−13.47^***^	10.11^***^	14.20^***^

Data are represented as mean ± SE. *t*-test values; ^***^ *P* < 0.001. ANOVA analysis: within each row, means with different superscript (a, b, c, or d) are significantly different at *P* < 0.05, whereas means superscripts with the same letters mean that there is no significant difference at *P* < 0.05. LSD: least significant difference.

**Table 4:** Effect of treating diabetic rats with *Moringa* seeds powder for 4 weeks on kidney functions and serum electrolytes.

Parameters mg/dL	Statistics	G1 −ve control	G2 +ve control	G3 50 mg *Moringa *	G4 100 mg *Moringa *
Blood urea nitrogen mg/dL	Mean ± SE LSD 0.05 = 4.479	23.83 ± 1.13^a^	68.33 ± 1.40^d^	52.50 ± 1.60^b^	34.16 ± 1.04^c^
	*t*-test		−20.49^***^	7.46^***^	14.31^***^

Creatinine mg/dL	Mean ± SE LSD 0.05 = 0.264	0.65 ± 0.04^a^	2.78 ± 0.15^d^	2.08 ± 0.06^b^	1.53 ± 0.04^c^
	*t*-test		−13.46^***^	4.18^***^	7.87^***^

Uric acid mg/dL	Mean ± SE LSD 0.05 = 0.196	3.33 ± 0.06^c^	6.20 ± 0.10^a^	4.35 ± 0.04^b^	4.16 ± 0.04^b^
	*t*-test	−26.41^***^	—	25.73^***^	16.18^***^

Sodium mmol/L	Mean ± SE LSD 0.05 = 2.402	121.83 ± 0.65^a^	141.00 ± 0.81^d^	128.00 ± 0.68^c^	136.17 ± 0.70^b^
	*t*-test		18.90^***^	−5.04^***^	−13.59^***^

Potassium mmol/L	Mean ± SE LSD 0.05 = 0.158	3.40 ± 0.03^a^	4.76 ± 0.05^d^	3.80 ± 0.05^c^	4.18 ± 0.06^b^
	*t*-test		14.87^***^	−4.67^***^	−11.14^***^

Data are represented as mean ± SE. *t*-test values; ^***^ *P* < 0.001. ANOVA analysis: within each row, means with different superscript (a, b, c, or d) are significantly different at *P* < 0.05, whereas means superscripts with the same letters mean that there is no significant difference at *P* < 0.05. LSD: least significant difference.

**Table 5:** Effect of treating diabetic rats with *Moringa* seeds powder for 4 weeks on urine analysis.

Parameters	Statistics	G1 −ve control	G2 +ve control	G3 50 mg *Moringa *	G4 100 mg *Moringa *
U. glucose mg/d	Mean ± SE LSD 0.05 = 8.085	20.91 ± 1.46^a^	219.72 ± 4.22^d^	113.78 ± 0.68^b^	81.70 ± 2.29^c^
	*t*-test		−41.04^***^	28.86^***^	23.04^***^

U. creatinine mg/d	Mean ± SE LSD 0.05 = 3.268	26.33 ± 0.61^a^	80.50 ± 0.61^d^	58.33 ± 1.22^b^	38.50 ± 1.54^c^
	*t*-test		68.36^***^	−27.04^***^	−6.21^***^

U. urea nitrogen mg/d	Mean ± SE LSD 0.05 = 36.846	123.00 ± 3.39^a^	627.00 ± 24.13^d^	217.83 ± 1.07^c^	417.50 ± 3.63^b^
	*t*-test		21.38^***^	−31.33^***^	−50.61^***^

U. albumin mg/dL	Mean ± SE LSD 0.05 = 15.750	40.97 ± 9.98^a^	193.30 ± 0.33^d^	181.67 ± 5.86^b^	117.50 ± 2.87^c^
	*t*-test		−39.36^***^	15.03^***^	30.91^***^

U. sodium mEq/L/day	Mean ± SE LSD 0.05 = 2.891	36.00 ± 0.51^a^	62.83 ± 1.07^d^	51.16 ± 0.94^b^	42.16 ± 1.13^c^
	*t*-test		−20.61^***^	7.67^***^	10.72^***^

U. potassium mEq/L/day	Mean ± SE LSD 0.05 = 2.075	71.16 ± 0.70^a^	98.00 ± 0.89^d^	88.83 ± 0.90^b^	81.66 ± 0.66^c^
	*t*-test		−33.86^***^	7.04^***^	22.84^***^

Data are represented as mean ± SE. *t*-test values; ^***^ *P* < 0.001. ANOVA analysis: within each row, means with different superscript (a, b, c, or d) are significantly different at *P* < 0.05, whereas means superscripts with the same letters mean that there is no significant difference at *P* < 0.05. LSD: least significant difference.

**Table 6:** Effect of treating diabetic rats with *Moringa* seeds powder for 4 weeks on total body weight.

Total body weight (g)	Statistics	G1 −ve control	G2 +ve control	G3 50 mg *Moringa *	G4 100 mg *Moringa *
Initial weight	Mean ± SE LSD 0.05 = 11.484	191.83 ± 1.68^a^	194.50 ± 1.78^a^	197.83 ± 2.02^a^	198.83 ± 2.32^a^
	*t*-test		−0.94^NS^	−1.28^NS^	−1.69^NS^

1st week	Mean ± SE LSD 0.05 = 9.690	195.00 ± 1.03^b^	170.83 ± 2.07^c^	194.50 ± 0.88^b^	206.50 ± 0.71^a^
	*t*-test		15.30^***^	−14.93^***^	−21.66^***^

2nd week	Mean ± SE LSD 0.05 = 3.000	202.67 ± 0.95^b^	173.83 ± 1.32^c^	202.50 ± 0.76^b^	210.67 ± 0.42^a^
	*t*-test		13.50^***^	−20.04^***^	−26.76^***^

3rd week	Mean ± SE LSD 0.05 = 3.107	207.83 ± 0.70^b^	163.33 ± 1.40^c^	210.17 ± 0.54^b^	215.00 ± 0.77^a^
	*t*-test		23.73^***^	−26.341^***^	−30.635^***^

4th week	Mean ± SE LSD 0.05 = 6.041	213.67 ± 1.58^a^	148.33 ± 2.85^b^	218.17 ± 0.94^b^	225.17 ± 1.13^b^
	*t*-test		14.893^***^	−22.053^***^	−24.592^***^

Data are represented as mean ± SE. *t*-test values; ^***^ *P* < 0.001. ANOVA analysis: within each row, means with different superscript (a, b, c, or d) are significantly different at *P* < 0.05, whereas means superscripts with the same letters mean that there is no significant difference at *P* < 0.05. LSD: least significant difference.

**Table 7:** Effect of treating diabetic rats with *Moringa* seeds powder for 4 weeks on water consumption.

Water consumed mL/day	Statistics	G1 −ve control	G2 +ve control	G3 50 mg *Moringa *	G4 100 mg *Moringa *
1st week	Mean ± SE LSD 0.05 = 3.257	33.33 ± 1.05^a^	42.50 ± 1.11^b^	36.33 ± 0.88^b^	36.33 ± 0.88^b^
	*t*-test		−4.56^***^	7.40^***^	4.01^***^

2nd week	Mean ± SE LSD 0.05 = 2.308	33.66 ± 0.88^c^	42.50 ± 1.11^a^	34.83 ± 0.90^c^	37.16 ± 0.79^b^
	*t*-test		−10.60^***^	5.56^***^	4.54^***^

3rd week	Mean ± SE LSD 0.05 = 3.431	29.16 ± 1.53^b^	42.50 ± 1.11^a^	26.66 ± 1.66^c^	26.66 ± 1.05^c^
	*t*-test		−8.00^***^	7.88^***^	19.00^***^

4th week	Mean ± SE LSD 0.05 = 3.832	27.50 ± 1.11^b^	41.33 ± 1.17^a^	28.00 ± 1.00^b^	27.50 ± 1.11^b^
	*t*-test		−6.43^***^	10.39^***^	6.67^***^

Data are represented as mean ± SE. *t*-test values; ^***^ *P* < 0.001. ANOVA analysis: within each row, means with different superscript (a, b, c, or d) are significantly different at *P* < 0.05, whereas means superscripts with the same letters mean that there is no significant difference at *P* < 0.05. LSD: least significant difference.

**Table 8:** Effect of treating diabetic rats with *Moringa* seeds powder for 4 weeks on food intake (FI) body weight gain (BWG) and food efficiency ratio (FER).

Biological evaluation parameters	Statistics	G1 −ve control	G2 +ve control	G3 50 mg *Moringa *	G4 100 mg *Moringa *
FI g/day	Mean ± SE LSD 0.05 = 0.387	16.83 ± 0.38^a^	16.20 ± 0.30^b^	15.95 ± 0.23^b^	15.95 ± 0.25^b^
	*t*-test		3.71^***^	1.29^***^	1.54^***^

BWG g/4 weeks	Mean ± SE LSD 0.05 = 6.031	28.66 ± 1.58^a^	−41.66 ± 2.85^c^	17.83 ± 0.94^b^	13.16 ± 1.13^b^
	*t*-test		16.03^***^	−19.92^***^	−17.55^***^

BWG %	Mean ± SE LSD 0.05 = 3.157	15.49 ± 0.85^a^	−21.92 ± 1.50^c^	8.90 ± 0.47^b^	6.20 ± 0.53^b^
	*t*-test		16.06^***^	−19.80^***^	−17.36^***^

FER g/day	Mean ± SE LSD 0.05 = 0.013	0.056 ± 0.003^a^	0.057 ± 0.029^c^	0.036 ± 0.001^b^	0.026 ± 0.002^b^
	*t*-test		3.80^**^	−3.09^**^	−2.85^***^

FER %	Mean ± SE LSD 0.05 = 1.433	5.61 ± 0.31^a^	−8.66 ± 0.66^c^	3.66 ± 0.18^b^	2.66 ± 0.24^b^
	*t*-test		14.97^***^	−17.63^***^	−15.79^***^

Data are represented as mean ± SE. *t*-test values; ^***^ *P* < 0.001 and ^**^ *P* < 0.01. ANOVA analysis: within each row, means with different superscript (a, b, c, or d) are significantly different at *P* < 0.05, whereas means superscripts with the same letters mean that there is no significant difference at *P* < 0.05. LSD: least significant difference.

**Table 9:** Effect of treating diabetic rats with *Moringa* seeds powder for 4 weeks on organs weight and relative organs weight.

Organ weight and relative organ weight	Statistics	G1 −ve control	G2 +ve control	G3 50 mg *Moringa *	G4 100 mg *Moringa *
Heart	Mean ± SE LSD 0.05 = 0.184	0.583 ± 0.060^a^	0.650 ± 0.071^a^	0.666 ± 0.066^a^	0.633 ± 0.033^a^
	*t*-test		−0.698^NS^	−0.155^NS^	0.191^NS^

Liver	Mean ± SE LSD 0.05 = 2.088	7.516 ± 0.245^a^	8.516 ± 0.305^a^	6.9167 ± 0.556^a^	6.583 ± 1.288^a^
	*t*-test		−5.085^NS^	4.393^NS^	1.366^NS^

Right kidney	Mean ± SE LSD 0.05 = 0.129	0.633 ± 0.055^a^	0.716 ± 0.016^a^	0.666 ± 0.046^a^	0.700 ± 0.044^a^
	*t*-test		−1.536^NS^	1.168^NS^	0.349^NS^

Left kidney	Mean ± SE LSD 0.05 = 0.121	0.816 ± 0.070^a^	0.666 ± 0.021^a^	0.633 ± 0.033^a^	0.683 ± 0.060^a^
	*t*-test		2.236^NS^	1.000^NS^	−0.277^NS^

Right testis	Mean ± SE LSD 0.05 = 0.214	1.133 ± 0.076^a^	1.316 ± 0.060^a^	1.133 ± 0.061^a^	1.166 ± 0.071^a^
	*t*-test		−1.437^NS^	4.568^NS^	2.423^NS^

Left testis	Mean ± SE LSD 0.05 = 0.192	1.166 ± 0.055^a^	1.266 ± 0.033^a^	1.133 ± 0.071^a^	1.250 ± 0.061^a^
	*t*-test		−1.168^NS^	1.865^NS^	0.277^NS^

Relative heart weight %	Mean ± SE LSD 0.05 = 0.111	0.268 ± 0.028^a^	0.438 ± 0.058^b^	0.300 ± 0.035^b^	0.276 ± 0.013^b^
	*t*-test		−2.584^**^	2.046^*^	2.662^**^

Relative liver weight %	Mean ± SE LSD 0.05 = .985	3.511 ± 0.115^a^	5.740 ± 0.196^b^	3.168 ± 0.113^b^	2.921 ± 0.573^b^
	*t*-test		−16.042^***^	11.594^***^	3.935^**^

Relative right kidney weight %	Mean ± SE LSD 0.05 = 0.067	0.281 ± 0.026^a^	0.480 ± 0.019^b^	0.301 ± 0.019^b^	0.305 ± 0.020^b^
	*t*-test		−6.327^***^	7.473^***^	6.220^***^

Relative left kidney weight %	Mean ± SE LSD 0.05 = 0.056	0.346 ± 0.018^a^	0.445 ± 0.016^b^	0.286 ± 0.014^c^	0.298 ± 0.027^bc^
	*t*-test		−4.709^***^	8.552^***^	5.526^***^

Relative right testis weight %	Mean ± SE LSD 0.05 = 0.116	0.525 ± 0.035^a^	0.885 ± 0.047^b^	0.515 ± 0.028^b^	0.515 ± 0.035^b^
	*t*-test		−4.666^***^	10.288^***^	9.081^***^

Relative left testis weight %	Mean ± SE LSD 0.05 = 0.097	0.540 ± 0.026^a^	0.850 ± 0.029^b^	0.516 ± 0.031^a^	0.550 ± 0.030^b^
	*t*-test		−5.901^***^	9.277^***^	7.729^***^

Data are represented as mean ± SE. *t*-test values; ^*^ *P* < 0.05, ^**^ *P* < 0.01, ^***^ *P* < 0.001. ANOVA analysis: within each row, means with different superscript (a, b, c, or d) are significantly different at *P* < 0.05, whereas means superscripts with the same letters mean that there is no significant difference at *P* < 0.05. LSD: least significant difference.

## Abbreviations

ALP: Serum alkaline phosphatase
ALT: Serum alanine aminotransferase
AST: Serum aspartate aminotransferase
b.w.: Body weight
BUN: Blood urea nitrogen
Cat: Catalase
Cr: Creatinine
DM: Diabetes mellitus
DN: Diabetic nephropathy
G1: Negative control group
G2: Positive control group
G3: Diabetic rats treated with 50 mg* Moringa*/kg b.w.
G3: Diabetic rats treated with 100 mg* Moringa*/kg b.w.
GSH: Reduced glutathione
HbA_1c_: Glycosylated hemoglobin
IL-6: Interleukin-6
SOD: Activity of superoxide dismutase
STZ: Streptozotocin.

## References

[1] Mahmood D., Singh B. K., Akhtar M. (2009). Diabetic neuropathy: therapies on the horizon. *Journal of Pharmacy and Pharmacology*.

[2] Shelbaya S., Amer H., Seddik S. (2012). Study of the role of interleukin-6 and highly sensitive C-reactive protein in diabetic nephropathy in type 1 diabetic patients. *European Review for Medical and Pharmacological Sciences*.

[3] Kanwar Y. S., Wada J., Sun L. (2008). Diabetic nephropathy: mechanisms of renal disease progression. *Experimental Biology and Medicine*.

[4] Mahajan S. G., Mali R. G., Mehta A. A. (2007). Protective effect of ethanolic extract of seeds of *Moringa oleifera* Lam. against inflammation associated with development of arthritis in rats. *Journal of Immunotoxicology*.

[5] Hamza A. A. (2010). Ameliorative effects of *Moringa oleifera* Lam seed extract on liver fibrosis in rats. *Food and Chemical Toxicology*.

[6] Yassa H. D., Tohamy A. F. (2014). Extract of *Moringa oleifera* leaves ameliorates streptozotocin-induced *Diabetes mellitus* in adult rats. *Acta Histochemica*.

[7] Mbikay M. (2012). Therapeutic potential of *Moringa oleifera* leaves in chronic hyperglycemia and dyslipidemia: a review. *Frontiers in Pharmacology*.

[8] Karadi R. V., Gadge N. B., Alagawadi K. R., Savadi R. V. (2006). Effect of *Moringa oleifera* Lam. Root-wood on ethylene glycol induced urolithiasis in rats. *Journal of Ethnopharmacology*.

[9] Makkar H. P. S., Francis G., Becker K. (2007). Bioactivity of phytochemicals in some lesser-known plants and their effects and potential applications in livestock and aquaculture production systems. *Animal*.

[10] Kumbhare M. R., Guleha V., Sivakumar T. (2012). Estimation of total phenolic content, cytotoxicity and in-vitro antioxidant activity of stem bark of *Moringa oleifera*. *Asian Pacific Journal of Tropical Disease*.

[11] Rajanandh M. G., Satishkumar M. N., Elango K., Suresh B. (2012). *Moringa oleifera* Lam. A herbal medicine for hyperlipidemia: a preclinical report. *Asian Pacific Journal of Tropical Disease*.

[12] Lambole V., Kumar U. (2012). Effect of *Moringa oleifera* Lam. on normal and dexamethasone suppressed wound healing. *Asian Pacific Journal of Tropical Biomedicine*.

[13] Asare G. A., Gyan B., Bugyei K. (2012). Toxicological evaluation of the aqueous leaf extract of *Moringa oleifera* Lam. (*Moringaceae*). *Journal of Ethnopharmacology*.

[14] Akbarzadeh A., Norouzian D., Mehrabi M. R. (2007). Induction of diabetes by Streptozotocin in rats. *Indian Journal of Clinical Biochemistry*.

[15] Srivastava L. M., Das N., Sinha S. (2002). *Essentials of Practical Biochemistry*.

[16] Schumann G., Klauke R. (2003). New IFCC reference procedures for the determination of catalytic activity concentrations of five enzymes in serum: preliminary upper reference limits obtained in hospitalized subjects. *Clinica Chimica Acta*.

[17] Tietz N. W., Shuey D. F. (1986). Reference intervals for alkaline phosphatase activity determined by the IFCC and AACC reference methods. *Clinical Chemistry*.

[18] Drury R. A., Wallington E. A., Cancerson R. (1976). *Carlton’s Histopathological Techniques*.

[19] Statistical Analysis System (SAS) (1986). *SAS User’s Guide: Statistics Version 5*.

[20] Szkudelski T. (2001). The mechanism of alloxan and streptozotocin action in B cells of the rat pancreas. *Physiological Research*.

[21] Ardawi M. S. M., Nasrat H. A. N., Bahnassy A. A. (1994). Serum immunoglobulin concentrations in diabetic patients. *Diabetic Medicine*.

[22] Vongsak B., Sithisarn P., Mangmool S., Thongpraditchote S., Wongkrajang Y., Gritsanapan W. (2013). Maximizing total phenolics, total flavonoids contents and antioxidant activity of *Moringa oleifera* leaf extract by the appropriate extraction method. *Industrial Crops and Products*.

[23] Ghiridhari W. A., Malhati D., Geetha K. (2011). Anti-diabetic properties of drumstick (*Moringa oleifera*) leaf tablets. *International Journal of Health and Nutrition*.

[24] Kasolo J. N., Bimenya G. S., Ojok L., Ochieng J., Ogwal-Okeng J. W. (2010). Phytochemicals and uses of *Moringa oleifera* leaves in Ugandan rural communities. *Journal of Medicinal Plants Research*.

[25] Lako J., Trenerry V. C., Wahlqvist M., Wattanapenpaiboon N., Sotheeswaran S., Premier R. (2007). Phytochemical flavonols, carotenoids and the antioxidant properties of a wide selection of Fijian fruit, vegetables and other readily available foods. *Food Chemistry*.

[26] Amaglo N. K., Bennett R. N., Lo Curto R. B. (2010). Profiling selected phytochemicals and nutrients in different tissues of the multipurpose tree *Moringa oleifera* L., grown in Ghana. *Food Chemistry*.

[27] Sayed A. A. R. (2012). Ferulsinaic acid modulates SOD, GSH, and antioxidant enzymes in diabetic kidney. *Evidence-Based Complementary and Alternative Medicine*.

[28] Scheller J., Chalaris A., Schmidt-Arras D., Rose-John S. (2011). The pro- and anti-inflammatory properties of the cytokine interleukin-6. *Biochimica et Biophysica Acta—Molecular Cell Research*.

[29] Kristiansen O. P., Mandrup-Poulsen T. (2005). Interleukin-6 and diabetes: the good, the bad, or the indifferent?. *Diabetes*.

[30] Awartani F. (2010). Serum immunoglobulin levels in type 2 diabetes patients with chronic periodontitis. *The Journal of Contemporary Dental Practice*.

[31] Anwar F., Latif S., Ashraf M., Gilani A. H. (2007). *Moringa oleifera*: a food plant with multiple medicinal uses. *Phytotherapy Research*.

[32] Afshari A. T., Shirpoor A., Farshid A. (2007). The effect of ginger on diabetic nephropathy, plasma antioxidant capacity and lipid peroxidation in rats. *Food Chemistry*.

[33] Ndong M., Uehara M., Katsumata S.-I., Suzuki K. (2007). Effects of oral administration of *Moringa oleifera* Lam on glucose tolerance in Goto-Kakizaki and wistar rats. *Journal of Clinical Biochemistry and Nutrition*.

[34] Parikh N. H., Parikh P. K., Kothari C. (2014). Indigenous plant medicines for health care: treatment of Diabetes mellitus and hyperlipidemia. *Chinese Journal of Natural Medicines*.

